# Salt in Agriculture
*On the Function of Salt in Agriculture. By A. Beauchamp Northcote, Esq., Senior Assistant in the Royal College of Chemistry. London: 1856.


**Published:** 1856-04

**Authors:** 


### SALT IN AGRICULTURE.*

Mr. Northcote, in inquiring into the use of salt in agriculture, arrives at the conclusion that "agricultural salt is a most energetic absorbent of ammonia; but that, at the same time, its agency does not seem to be altogether a permanent one: it will collect the ammonia, but it is questionable whether it can retain it for any great length of time, because, in the very decompositions which happen in order to render the ammonia more stable, salts are formed which have a direct tendency to liberate ammonia from its more fixed combinations. It may, however, retain it quite long enough for agricultural purposes: if the young plants are there ready to receive it, its state of gradual liberation may be for them the most advantageous possible; and to this conclusion all experiments on the large scale appear most obviously to tend. It is described as an excellent check to the too forcing power of guano; and from M. Barral's experiment we see that it either prevents the too rapid eremacausis of the latter, or stores up the ammonia as it is formed. As a manure for growing crops, all experience and all theoretical considerations therefore show it to be most valuable."

These results regarding the power of salt in absorbing ammonia, while strictly original, are very important in a chemical sense. We hope the learned author will pursue this subject further, and in other directions.

---

* On the Function of Salt in Agriculture. By A. Beauchamp Northcote, Esq., Senior Assistant in the Royal College of Chemistry. London: 1856.

